# Preliminary Evidence of Differentially Induced Immune Responses by Microparticle-adsorbed LPS in Patients with Crohn’s Disease

**DOI:** 10.33696/immunology.4.152

**Published:** 2022

**Authors:** P Ashwood

**Affiliations:** 1 The Department of Medical Microbiology and Immunology, University of California Davis, California, USA

**Keywords:** Titanium dioxide, Lipopolysaccharide, IL-1β, TNF-α, Inflammation, Microparticles, Autism spectrum disorders, Schizophrenia

## Abstract

Inorganic microparticles are ubiquitous in the modern Western diet present as food additives and are actively scavenged by microfold (M) cells overlying human intestinal lymphoid aggregates. In Crohn’s disease (CD), inflammation is caused by the inability of the intestinal mucosa to sustain tolerance to gut luminal factors including bacteria and their by-products. Having large, highly charged surface areas dietary particles can avidly bind biomolecules such as lipopolysaccharide (LPS). The aim of this paper was to examine whether the dietary particle, titanium dioxide (TiO_2_), modified cellular immune responses to LPS differently in peripheral blood mononuclear cells (PBMC) from CD patients compared with healthy controls. Our data showed that LPS-associated particles predominantly stimulated release of IL-1β and induced concurrent cell death in peripheral monocytes following particle uptake in both health and disease. In addition, IL-1β release was increased more in CD patients compared with controls following particle stimulation. In conclusion, LPS adsorption to dietary particulates provides a mechanism for stimulation of phagocytic mononuclear cells and may cause aggravation of mucosal immune responses in inflammatory conditions of the bowel such as CD, irritable bowel syndrome, and autism spectrum disorder and schizophrenia associated gastrointestinal conditions, by immune priming mediated through increased production of pro-inflammatory cytokines.

## Introduction

There is growing evidence that exposure of mucosal surfaces to particulate matter, for example in the lungs to particles present in air pollution, is associated with increased morbidity, mortality, or exacerbation of existing diseases. Both short- and long-term exposure to particulate air pollution have been linked to increased respiratory morbidity, and indeed even at low levels particulate air pollution has potentially adverse effects on health [1–6]. Increased vehicle use and the emission of exhaust fumes and thus particulate material, parallel’s the increase in childhood atopy [7,8]. So far, the biologic mechanisms behind these health effects remain unclear but are often more pronounced in susceptible population groups such as those with pre-existing respiratory conditions [9,10].

Fine particles can act as mucosal adjuvants, enhancing immune responses, such as the elevation of IgE titers to common allergens [11]. Conjugation of protein to particle surfaces and subsequent effects on cellular processing and presentation of antigen have been well studied. Environmental particles generally have large negatively charged surfaces that can facilitates the adsorption of macromolecules such as self-proteins, exogenous antigens, and bacterial proteins, onto their surfaces [12]. For example, fine particles from diesel exhaust fumes can adsorb several antigens, such as the grass pollen major allergen, Lol p1 [13]. In contrast, the conjugation of particles and bacterial components and their influences on cellular responsiveness have been less well investigated.

Substantial particle uptake occurs in lymphoid-rich areas of the human gastrointestinal tract and may be involved in exacerbation of inflammatory bowel disease. In the Western world, about 10^12^ particles of titanium dioxide are ingested per person/per day, and about 10^10^ are absorbed. The interaction of dietary particles with endogenous bacterial products and mucosal secretions of the gastrointestinal tract have not been studied, but LPS, for example, is abundant and it avidly binds to particle surfaces, such as titanium dioxide with calcium bridging cations [12,14,15]. Previously, we showed that particles of food additive-grade titanium dioxide amplify calcium and LPS-induced IL-1β secretion of intestinal cells [16].

Evidence from both animal and human studies implicate a dysregulated or exaggerated immune response is responsible for the disease pathogenesis in Crohn’s disease (CD) [17]. In genetically susceptible individuals an inappropriate immune response to commensal intestinal bacteria or their by-products, such as LPS, may play a prominent role in CD. Phagocytic cells are present beneath the intestinal epithelium and represent a first line of defence against luminal antigens. In actively inflamed CD lesions, there is a distinct CD14 positive population of recently recruited monocyte-like macrophages, which are more reactive than resident macrophages, releasing pro-inflammatory cytokines such as tumour necrosis factor-α (TNF-α) and interleukin-1β (IL-1β) [18–20]. Adsorption of LPS onto particles may provide a vehicle for the entry of bacterial by-products into the mucosa, inducing cellular activation in recently recruited macrophages and the release of pro-inflammatory cytokines following phagocytosis of the particles. Moreover, genetic predisposition of CD patients towards inflammation may lead to increased further pro-inflammatory cytokine release after particle ingestion. Furthermore, there may be more widespread implications of the effects of particles in newly emerging conditions where innate inflammation and gastrointestinal inflammation exist, such as autism spectrum disorders and schizophrenia [21–26]. Indeed, mucosal responses to titanium dioxide (TiO_2_) have been linked to causation of autism behaviours in an animal model [27] and dietary interventions can alleviate autism symptoms [23].

The aim of this study was to compare the responses to LPS stimulation in the presence of particles in health and disease. The responses of peripheral blood monocytes from CD patients, which mimics the CD14 positive cells found in IBD mucosa [28], to a conjugate of LPS and the ubiquitous food additive TiO_2_, in the presence of an ion bridge, (the LPS-Ca^2+^-TiO_2_ conjugate) were compared with monocytes obtained from normal healthy volunteer donors.

## Methods

### Research participants

All procedures involving participants were approved by the Ethics Committee at St Thomas’ Hospital and all subjects signed informed consent. Peripheral blood was collected from 16 patients (mean age 40 ± 5; 8 female) with well characterised mild to moderate CD who were consecutively attending out-patient clinics at St. Thomas’ Hospital and were compared with 12 healthy volunteers (mean age 38 ± 4; 6 female) who were attending wellness visits. Volunteers were excluded if they showed any signs of illness (e.g. common cold, viral infection, allergy, etc) or were currently on medication. Whole blood from each subject was diluted 1:1 in saline before peripheral blood mononuclear cells (PBMC) were isolated by density gradient centrifugation over Lymphoprep^™^ (Nycomed, Oslo, Norway). The interface was collected, and the cells were washed and resuspended in tissue culture medium (TCM) comprising of RPMI 1640 medium containing, 10 μg/ml gentamicin (Sigma), 200 mM L-glutamine (Sigma), 100U/ml penicillin (Sigma), 100 μg/ml streptomycin (Sigma) and 10% FCS (Gibco). Cell number and viability was assessed by 0.4 % trypan blue (Sigma).

### Incubation with test stimulants

PBMC (1 × 10^6^ cells) were treated with varying concentrations of either LPS (0.1–100 ng/ml) (Sigma, UK), TiO_2_ (1–20 μg/ml) (Tioxide Ltd., UK) or CaCl_2_ (40–800 μg/ml of Ca^2+^) (BDH, UK) for 24h at 37°C in 5% CO_2_ and 95% air. In addition, a conjugate of LPS-Ca^2+^-TiO_2_ conjugate (final concentrations 1 ng/ml LPS plus 5 μg/ml TiO_2_ plus 160 μg/ml Ca^2+^) was also tested and prepared by vortex mixing and sonication before addition to PBMC. All test solutions were prepared in ultrapure sterile water. Results were compared to control samples treated only with the same volume of ultrapure sterile water. Further PBMC cultures were treated for differing incubation times of 4, 24, and 48 h, in the presence of either (i) 1 ng/ml LPS alone, (ii) 1 ng/ml LPS plus 5 μg/ml TiO_2_, (iii) 1 ng/ml LPS plus 160 μg/ml Ca^2+^ (as CaCl_2_) or (iv) the LPS-Ca^2+^-TiO_2_ conjugate. At the end of each incubation period the supernatants were removed following centrifugation and stored at −70°C prior to IL-1β measurements. Cells were processed for analysis of propidium iodide (PI) incorporation and CD14 expression and by flow cytometry. Previously we have shown that increased cell death and increased IL-1β release are the most discriminating effect of the particle stimulation in mucosal monocytes, and so these outcome measures were assessed here [12,16,29]. Apoptotic events were detected by the PI incorporation into the sub G0 peak.

Assessment of basal or stimulated IL-1β release by PBMC was performed by enzyme-linked immunosorbent assay (ELISA) kits (R & D, UK) according to manufacturer’s instruction (sensitivity of <1 pg/ml). Single cell suspensions suitable for flow cytometry were prepared for analysis of cell surface CD14 expression and apoptosis by PI incorporation. Following culture, cells were washed twice in TCM, prior to staining with mouse antihuman monoclonal CD14-FITC (Serotec, UK) in the residual cell suspension (200 μl cells in TCM) for 30 mins on ice in the dark. After staining cells were washed three times in TCM, to remove non-specific binding, before fixation in 2 ml 70% ethanol/30% ultrapure water and stored overnight at 4°C in the dark. Overnight incubation in 70% ethanol permits leakage from the cell of small fragments of DNA which are generated during apoptosis. Propidium iodide intercalates with DNA, and reduced DNA content can be visualised as an increase in staining in the subGO peak. Prior to PI staining, cells were checked for microscopic clumping and if necessary, passed through a sterile 25-gauge needle. 100 μl ribonuclease solution (1 mg/ml) (Sigma) and 100 μl propidium iodide (400 mg/ml) (Sigma) were added to the cells and incubated at 37°C in 5% CO_2_ and 95% air for 30 min. PBMC were analysed within 1 h of PI staining using a FACScan equipped with a 480nm laser (Becton and Dickinson). Measurements of forward (FSC) and side scatter (SSC) plus CD14 FITC-fluorescence (FL1) and PI fluorescence (FL2) were recorded.

Physical characteristics of cells based on size (forward scatter) and granularity (side scatter) properties were used to generate a gated region that excluded lymphocytes and debris but included monocytes. Using this defined region CD14 expression (exclusively present within the monocyte gate) and PI incorporation into the sub G0 peak between untreated and treated samples was compared. 10,000 events were obtained per sample and data is represented as percentage of total events.

### Particulate calcium formation

Calcium will form particulates in phosphate and/or carbonate containing tissue culture media, the formation of these calcium particles was examined in two different aqueous cell culture media. TCM was prepared as before using RPMI 1640 (phosphate rich) as the basis, while in addition, TCM was prepared using Dulbecco’s modified Eagle’s medium (DM, phosphate poor). In both culture media the same supplements (i.e. gentamicin, L-glutamine, penicillin, streptomycin) were used at identical concentrations. Control PBMC were resuspended in either TCM at 5 × 10^5^ cells/ml. 160 μg/ml Ca^2+^ ions, in the form of CaC0_3_, CaCl_2_ or CaPO_4_ were added separately to cells prepared in either RPMI or DM based TCM. The incorporation of PI into the sub G0 peak, was examined after 24 h incubation of cells at 37°C in 5% CO_2_ and 95% air and compared to LPS-Ca^2+^-TiO_2_ induced cell death.

### Statistical analysis

The effects of stimulation with the individual components comprising the LPS-Ca^2+^-TiO_2_ conjugate were analysed over dose ranges. Responses of control PBMC to stimulation were compared with those from CD PBMC. Stimulation within each sample group was analysed using the paired Student’s T-test after testing for normality of distribution and performed at concentrations that elicited maximal responses. Comparisons between sample groups were performed, following correction for variance in the baseline, using the Mann-Whitney U test. Finally, particulate induced cell death was assessed in PBMC cultures, prepared in media containing either high or low levels of phosphate. To account for differing baseline levels of cell death, stimulated ratios (treated: untreated) were used. All results are expressed as means ± S.E.M. Statistics was performed using SPSS software.

## Results

### Soluble LPS induced stimulation

The release of IL-1β from PBMC obtained from healthy controls and CD patients stimulated with varying concentrations of LPS reached a plateau after addition of 1 ng/ml LPS (p < 0.01, LPS (1 ng/ml) vs. controls, for both patient groups). Baseline levels of IL-1β released from CD patients (613.2 ± 117.8 pg/ml) were significantly higher than controls (117.9 ± 51.4 pg/ml, p=0.008) and this difference was maintained throughout the dose response curve (Table 1). The number of untreated monocytes expressing the cell surface antigen CD14 was generally higher in PBMC from healthy controls compared to CD patients. No significant changes in CD14 expression were seen on increasing doses of LPS in either group, although the trend towards higher expression was maintained in the normal volunteers (Table 1). Propidium Iodide incorporation into the hypoploidy (Sub G0) peak was unchanged with increasing doses of LPS in PBMC from both healthy control and CD patients. As seen with IL-1β baseline responses of PBMC from CD patients showed elevated PI incorporation compared to healthy controls (17.3 ± 2.0 vs. 10.4 ± 1.3%, p=0.03). The differences in PI incorporation between CD patients and healthy control cells were maintained throughout the LPS dose response curve (Table 1).

### Response to TiO_2_ treatment

Following challenge with TiO_2_, IL–1β release from healthy control PBMC was initially elevated with low dose TiO_2_ (1 μg/ml), but no further increases were observed at higher doses. In PBMC from CD patients there was an initial increase in IL-1β release following challenge with TiO_2_ (Table 1), which peaked at 5 μg/ml TiO_2_, with 12 out of 16 CD patients showing elevated release of IL-1β following treatment with 5 μg/ml TiO_2_ and was significantly increased compared with controls (p<0.01). CD14 expression was markedly decreased in both healthy control and CD PBMC following challenge with TiO_2_ (p<0.01, for responses at 5 μg/ml TiO_2_). The generally lower baseline level of CD14 expression in CD patients compared to controls was mirrored throughout the dose response. This decreased CD14 may indirectly indicate increased apoptosis or membrane switching after phagocytosis. Annexin V staining as a marker of apoptosis in monocytes is confounded due to a large degree of membrane switching after phagocytosis. However, PI incorporation as a measure of apoptosis, was increased into healthy control PBMC with increasing doses of TiO_2_. In CD patients there was a similar increase in PI incorporation with increasing TiO_2_ dose, which reached a plateau between 5 and 10 μg/ml TiO_2_. PI incorporation in both groups was significantly different from baseline levels at 10 μg/ml TiO_2_ (p<0.02). Furthermore, loss of CD14 expression inversely paralleled PI incorporation (Table 1).

### Stimulation with CaCl_2_

A steady rise in IL-1β release was seen with increasing doses of CaCl_2_ (Table 1). The response reaching a plateau after addition of 160 μg/ml CaCl_2_ in both healthy control and CD PBMC and was increased in CD compared to controls (p<0.02). Responses were similar, but more marked increases were observed in PBMC from CD patients compared to healthy control samples. Indeed, after correcting for baseline values of IL-1β release, the response to CaCl_2_ (160 μg/ml of Ca^2+^) was significantly higher in CD patients compared to control subjects (2388 ± 519 vs. 788 ± 282 pg/ml, baseline corrected values for CD vs. normal IL-1β release, respectively, p=0.01). PI incorporation peaked at 160 μg/ml CaCl_2_ in PBMC from both control and CD subjects and was increased in CD compared to controls (p<0.01) and was followed by a sharp fall in PI incorporation at higher doses of CaCl_2_ (Table 1). However, baseline corrected responses to CaCl_2_ were not significantly different between CD and normal subjects (e.g. at 160 μg/ml Ca^2+^, PI incorporation was 29 ± 5 % v 25 ± 6 %, CD v normal, respectively). In both groups, CD14 expression inversely paralleled the response seen for PI incorporation, reaching a minimal response at 160 μg/ml CaCl_2_ (p<0.02 vs. controls).

### Responses to particles in presence of LPS

Responses to LPS (1 ng/ml) in the presence of CaCl_2_ (160 μg/ml as Ca^2+^) or TiO_2_ (5 μg/ml), was examined for differential responses between CD and normal PBMC. No additive effect was noted when PBMC were stimulated with LPS in the presence of TiO_2_. LPS plus TiO_2_ stimulated IL-1β release to levels similar to those seen for LPS alone at 24 h in both normal (1660 ± 672 pg/ml) and CD (2036 ± 786 pg/ml) PBMC. However, stimulation with LPS in the presence of CaCl_2_ elevated levels of IL-1β release at 24 h above LPS alone levels, from CD (10274 ± 1069 pg/ml) and normal subjects (4443 ± 936 pg/ml), with a significantly higher response in cells from CD patients compared with normal control PBMC (p=0.02). Time course experiments (2.5 h, 24 h, and 48 h) all showed the same trends and did not diverge in patient from control groups. Interestingly, TiO_2_ in presence of CaCl_2_ was not significantly different from the individual components (1187 ± 987 pg/ml vs. 2787 ± 815 pg/ml, for control vs. CD). Levels of PI incorporation induced by LPS plus TiO_2_ were constant over time in both normal and CD PBMC, with no significant differences in PI incorporation seen between the groups at 24 h following LPS plus TiO_2_ treatment (28.5 ± 6.6% vs. 32.3 ± 3.3%, CD vs. controls). LPS plus CaCl_2_ however, induced greater levels of PI incorporation (40.5 ± 6.1% vs. 33.3 ± 7.6%, CD vs. controls) than LPS plus TiO_2_ 48 h in CD but no significant differences were observed between the two groups. Similar reductions in expression of CD14 were observed in both CD and control PBMC following addition of LPS in the presence of TiO_2_ or CaCl_2_, with no significant differences noted between groups.

### The effect of the LPS-Ca^2+^-TiO_2_ conjugate

IL-1β release was increased equally following challenge with the LPS-Ca^2+^-TiO_2_ conjugate in both CD and control groups. Responses to the LPS-Ca^2+^-TiO_2_ conjugate were significantly increased in CD compared to control volunteers at 24 h (P = 0.018). Elevated IL-1β release was detected as early as 2.5 h incubation (1195 ± 744 pg/ml vs. 778 ± 469 pg/ml., CD vs. control) and peaked at 24 h (9636 ± 985 pg/ml vs. 4254 ± 1152 pg/ml, CD vs. control, p = 0.018). A corresponding increase in PI incorporation was observed after treatment with the LPS-Ca^2+^-TiO_2_ conjugate, again the response peaking at 24 h (44.8 ± 5.1% vs. 40.0 ± 4.7%, CD vs. controls). PI incorporation was inversely paralleled by a decrease in CD14 expression (4.7 ± 0.5% vs. 4.9 ± 1.2%, CD vs. controls). No differences in PI incorporation or CD14 responses to the LPS-Ca^2+^-TiO_2_ conjugate were observed between mononuclear cells isolated from CD or control subjects. Interestingly, the IL-1β response induced by CaCl_2_ in the presence of LPS was similar in CD patients to levels following stimulation with the LPS-Ca^2+^-TiO_2_ conjugate (10,274 ± 1069 pg/ml and 9636 ± 985 pg/ml, respectively). This finding was also present in cells from controls but at approximately decreased 2.5-fold the level. Based on this finding we investigated the effects of Ca^2+^ particulates in media.

### Calcium particle formation

Cell death (PI incorporation) in cultures of PBMC was shown to be the most discerning measure of particulate material stimulation. Indeed, TiO_2_ treatment alone was capable of inducing apoptosis in PBMC (Table 1). The effect of adding Ca^2+^ to media either enriched (RPMI) or low (DM) in phosphate was examined for the stimulation of cell death in control subjects. Addition of Ca^2+^ to PBMC from control volunteers prepared in RPMI based TCM had an increased baseline level of cell death compared to those prepared in DM based TCM (35 ± 6.8% and 7.8 ± 2.4%, respectively, p=0.001). In addition, CaPO_4_ treatment alone induced elevated cell death above baseline control levels in both DM and RPMI based TCM. Secondly, treatment with the CaCO_3_, which forms fewer particles compared to CaPO_4_, did not induce cell death in either of the media. Finally, we investigated the effects of particulate formation on IL-1 responses. The LPS-Ca^2+^-TiO_2_ conjugate or LPS-Ca^2+^ complex was prepared as before but centrifuged at 12,000 g for 10 min and the supernatants were then removed and used to stimulate PBMC cultures. Removal of particulates negated any IL-1β stimulatory effect (Table 2) suggesting particle formation was required for monocyte activation.

## Discussion

The intestinal mucosa is constantly exposed to large concentrations of LPS present on the outer membranes of resident Gram-negative bacteria. LPS is a potent immune stimulator capable at very low concentrations (picogram), of activating macrophages or monocyte derived dendritic cells to release pro-inflammatory cytokines. The close proximity of LPS and immune responsive mucosal cells, such as macrophages, located immediately beneath the epithelium or dendritic cells within the lamina propria, suggests that the risk for inflammatory responses to arise are potentially high. In all subjects analysed in this study, 1 ng/ml LPS was sufficient to induce the release of IL-1β from monocytes confirming previous studies [12,30,31]. The intrinsic capacity of CD patients to produce higher levels of cytokine secretion compared to normal healthy volunteers was also observed and reflects a putative genetic predisposition in CD patients for inappropriate immune responses [17]. Stimulation of PBMC from CD patients with TiO_2_ (5 μg/ml) resulted in a significant rise in IL-1β secretion, suggesting that particles from the diet might be of clinical relevance. These responses appear specific to CD patients, because similarly treating PBMC from healthy donor volunteers with TiO_2_ did not stimulate IL-1β release. Indeed, these data would seem to corroborate the findings that CD patients maintained on a diet low in particles (e.g. calcium and TiO_2_) showed a reduction in disease activity compared to CD patients kept on a more conventional diet [32].

Further evidence for an abnormal response to particulates in CD patients was seen for IL-1β secretion following addition of CaCl_2_. Our data showed that CaCl_2_ forms *de novo* particulates in phosphate rich medium that leads to increased apoptosis. This effect was negated by using low phosphate media and/or centrifugation of particulate matter. Stimulation of both normal control and CD PBMC with CaCl_2_ raised IL-1β levels. As Ca^2+^ plays a central role in many cellular processes the responses possibly may be due to an increase in cellular activity in already primed cells rather than an effect of particulates per se. Indeed, calcium is rapidly solubilised in the cell following uptake of calcium phosphate crystals by phagocytes [33] and leads to the induction of MAP kinase activation and subsequent production of reactive oxygen intermediates [34]. In addition, Ca^2+^ dependent cytokine release or activation of Ca^2+^-dependent endonucleases involved in the apoptosis process, may be a direct consequence of raised intracellular Ca^2+^ levels following particle uptake. Preliminary supportive evidence for such a process comes from the findings of increased cell death, assessed by PI incorporation, following CaCl_2_ stimulation. However, increased cell death was also seen in PBMC treated with TiO_2_ and may reflect intracellular signalling stimulated after phagocytosis. Cell death after treatment with TiO_2_ or CaCl_2_ was observed in both CD and normal control cells, with no differences between the groups.

Stimulation of PBMC with the LPS-Ca^2+^-TiO_2_ conjugate increased IL-1β secretion and apoptosis. We have previously shown that IL-1β, IL-6 and TNF-α are increased after stimulation with the LPS-Ca^2+^-TiO_2_ conjugate but that IL-10 is decreased, suggestion and imbalance in immune response [12]. These data may also suggest the activation of NF-κB and inflammasomes through LPS and particulates, respectively. Differential responses to the LPS-Ca^2+^-TiO_2_ conjugate in PBMC cultures from patients with CD disease compared to those from normal healthy subjects were also seen and was similar to stimulation with CaCl_2_ administered in the presence of LPS, in the context of CD but not controls. These finding may suggest that CD monocytes are maximally stimulated by both particulate complexes/conjugates whereas control monocytes are not. Alternatively, CD monocytes may be intrinsically primed to respond to particles following phagocytosis, whereas monocytes from control patients need an additional signal provided by TiO_2_ or LPS to exert similar responses. Indeed, uptake mechanisms may be enhanced in CD monocytes compared to controls volunteers and may explain their responses to all particulate groups. The induction of phagocytosis in CD and controls to particulates is worthy of further analysis. Further experiments to discern the pro-inflammatory nature of micro-particulates in the diet in health and disease needs to be confirmed in a separate, longer series of experiments.

Increased release of pro-inflammatory cytokines at the site of mucosal inflammation is a common feature of active IBD, with many cytokine levels correlating with disease severity. The pattern of released cytokine is characterised by a shift towards pro-inflammatory signals and away from competitive regulatory signals. Through this elaboration of the pro-inflammatory cytokine cascade, highly reactive cells are recruited into the mucosa, where their activation and stimulation may lead to the progression and perpetuation of the disease.

In the normal intestine, entry of environmental factors, including the commensal gut flora and dietary constituents, are restricted by physical barriers such as mucus, microvilli and the glycocalyx. However, one route of entry for putative environmental aetiological agents into the intestinal mucosa is through the intestinal Peyer’s patches (PP). These dome-like structures covered by specialised M-cells scavenge particulate material from the gut lumen and expedite their transport across the mucosal barrier. For example, particles of TiO_2_, which are present as food additives in the diet, adhere to M-cells and are conveyed to underlying macrophage where they accumulate in pigmented cells at the base of PP [35–37]. Inorganic particles may provide a vehicle with which bacterial products, such as LPS, may gain access to the mucosa. The delivery of such potent immunogenic material on particles may evoke immune responses in otherwise normally hyporesponsive mucosa. This may also be important in conditions with increased intestinal permeability and altered tight junction integrity such as in individuals with autism and schizophrenia [21–26] where particulates may facilitate the entry of bacteria into the lamina propria and away from the regulatory mechanisms generated in the PP.

Dietary TiO_2_ (in the form of anatase) is resistant to degradation in the gut lumen or the phagolysosomes of mucosal macrophages and survives for prolonged periods in pigment cells [35]. The physical and chemical properties of TiO_2_ permit adsorption of biologically active biomolecules, such as LPS, on to its surface [12,16,29]. The binding of such biomolecules is through electrostatic forces, where the Ca^2+^ ions interact with negatively charged oxide coatings on titanium particles and anionic sites present on the biomolecule, such as sulphate and carboxyl groups. The amount of TiO_2_ ingested per person per day is about 5 mg, while the level of Ca^2+^ ions present in the gastrointestinal lumen is 160 – 400 μg/ml. At these concentrations there is considerable scope for Ca^2+^-TiO_2_ interactions in the gut lumen and the subsequent binding of LPS derived from the degradation of the numerous resident Gram-negative bacteria.

In previous experiments analysis of apoptosis gave the most discriminating effects of particulate v soluble species [12]. In PBMC from control participants, cell death was observed after stimulation with the particle TiO_2_, but IL-1β release was not promoted. The analysis of the effects of CaCl_2_ in phosphate rich RPMI media showed an increase in apoptosis similar for the LPS-Ca^2+^-TiO_2_ conjugate or a particulate form of calcium, namely CaPO_4_. However, in media low in phosphate, no apoptosis was observed upon CaCl_2_ treatment. This confirms previous findings that Ca^2+^ from CaCl_2_ co-precipitates with phosphate forming calcium phosphate particles [38] capable of inducing apoptosis. Together with phosphate, high levels of carbonate are also present within the (RPMI) medium. However, when CaCO_3_ was added to cells there were no increase in apoptosis in either tissue culture media, showing that CaPO_4_, and not CaCO_3_, is the active, precipitating species when CaCl_2_ is added to RPMI. Furthermore, centrifugation of the particulate mixtures was sufficient to negate the effects on IL-1β release. We have also shown that solubilizing Ca^2+^ with citric acid also reduced particulate formation [16]. As part of the active LPS-Ca^2+^-TiO_2_ conjugate calcium is responsible for binding LPS, and acts as the ‘glue’ between TiO_2_ and LPS, but in a particulate form calcium was also shown to avidly bind LPS. Indeed, CaCl_2_ in the presence of LPS stimulated PBMC from CD patients but not those from normal volunteer IL-1β reaching similar levels to that achieved with the LPS-Ca^2+^-TiO_2_ conjugate. Together, these findings suggest that in CD there is an increased capacity for stimulation by particulates.

There are a number of limitations to this study including the small sample sizes. However, this study was a preliminary investigation that warrants further follow up in larger cohorts. In addition, in this study we examined responses in individuals with mild/moderate CD. Further studies with severe CD or a range of disease severity would help elucidate the responses to particulates and whether they had a bigger response in milder cases. We believe these initial studies provide a framework for future studies, not just in IBD but also in other GI conditions such as IBS, autism, or schizophrenia.

In conclusion there was a differential response when the LPS-Ca^2+^-TiO_2_ conjugate, was exposed to mononuclear cells from CD patients compared with healthy control subjects. These particulate mixtures caused a greater increase in IL-1β release from CD cell cultures than normal volunteer cells. Particulates formed due to the adsorption of LPS to calcium particles were also able to increase IL-1β secretion in CD patients and reached levels of stimulation similar to the LPS-Ca^2+^-TiO_2_ conjugate. This form of particulate calcium is likely to be CaPO_4_. Thus, particle stimulation by TiO_2_ or Ca^2+^ may be of clinical relevance in CD patients due the increase of dietary particulates in Western diets. Moreover, the presence of calcium or TiO_2_ in the Western diet may similarly cause aggravation of inflammation in the gastrointestinal tract in IBD and in other conditions where mucosal inflammation is a key co-morbidity such as in schizophrenia or autism spectrum disorders.

## Figures and Tables

**Table 1.** Stimulation of PBMC with LPS, TiO_2_ and CaCL_2_ dose response curves in Crohn’s disease and healthy controls, as individual components of the LPS-TiO2-Ca^2+^ conjugate.

		Baseline	LPS (ng/ml)			TiO_2_ (μg/ml)			CaCl_2_ (μg/ml)		
			0.1	1	100	1	5	10	80	160	320
**IL-1β (pg/ml)**	**Normal**	117.9 ± 51.4	452 ± 118.1	1102 ± 393.6	1280.6 ± 465.5	201 ± 58	155 ± 42	213 ± 99	646.7 ± 230.5	1080.6 ± 354.2	1265.6 ± 287.1
	**Crohn’s**	613.2± 117.8	1110.8 ± 452.1	1634.1 ± 619.5	1629.3 ± 834.4	649 ± 332.6	1114.6 ± 656	990.5 ± 345.5	1319 ± 400	3584 ± 803	2764 ± 976
**CD14 (%)**	**Normal**	44 ± 6	50.4 ± 6.7	40.9 ± 3.2	40.6 ± 2.6	45 ± 4.7	25.7 ± 7.7	16.9 ± 3.9	11.9 ± 4.9	8.1 ± 8	22.6 ± 4.1
	**Crohn’s**	33.5 ± 4	31 ± 5.3	30.3 ± 8.1	31.4 ± 7.4	19.2 ± 5.6	5.2 ± 1.5	5.4 ± 3.6	7.8 ± 4	3.5 ± 1.4	8.9 ± 2.7
**PI in Sub-(GO) (%)**	**Normal**	10.4 ± 1.3	10.5 ± 2.1	11.4 ± 1.5	7.3 ± 1.2	10.9 ± 2.1	15.8 ± 2.7	18.4 ± 2.9	15.3 ± 1.9	35 ± 6.8	17.6
	**Crohn’s**	17.3 ± 2	16.1 ± 2.1	16.3 ± 2.9	18.6 ± 2.7	23.3 ± 4	29.7 ± 5.2	33 ± 3.8	28.1 ± 8	48.7 ± 4	30.9 ± 2.3

**Table 2.** The effect of centrifugation on the ability of particle suspensions to stimulate IL-1β release (pg/ml).

	Centrifugation		
	Pre-	Post-	p values
**Control**	164 ± 103		
**LPS-Ca** ^**2+**^ **-TiO**_**2**_	3267 ± 939	968 ± 422	**0.02**
**LPS plus CaCl**_**2**_	929 ± 36	456 ± 153	**0.04**

# Test solutions of either the LPS-Ca^2+^-TiO_2_ conjugate or LPS-Ca^2+^ complex was prepared and centrifuged at 12,000 g for 10 min and the supernatants removed and added to PBMC (1 × 10^6^ cells) cultures. Parallel PBMC cultures were treated with identical solutions prepared without centrifugation. The effect of removing particulate material from the test solutions was compared for stimulated IL-1β release (pg/ml), detected by specific ELISA. Data are expressed as mean ± S.E.M. n= 4. Differences were considered significant if p<0.05 for paired Student’s t-test.
